# Mitochondrial Electron Transport Chain Protein Abnormalities Detected in Plasma Extracellular Vesicles in Alzheimer’s Disease

**DOI:** 10.3390/biomedicines9111587

**Published:** 2021-10-31

**Authors:** Pamela J. Yao, Erden Eren, Edward J. Goetzl, Dimitrios Kapogiannis

**Affiliations:** 1 Laboratory of Clinical Investigation, National Institute on Aging, 251 Bayview Blvd, Baltimore, MD 21224, USA; YaoPa@grc.nia.nih.gov (P.J.Y.); erden.eren@nih.gov (E.E.); Edward.Goetzl@ucsf.edu (E.J.G.); 2 Department of Medicine, University of California, San Francisco, CA 94143, USA; 3 San Francisco Campus for Jewish Living, San Francisco, CA 94112, USA

**Keywords:** NADH, oxidative phosphorylation, Alzheimer’s disease, mitochondria, electron transport chain, Superoxide Dismutase 1, SOD1

## Abstract

Mitochondria provide energy to neurons through oxidative phosphorylation and eliminate Reactive Oxygen Species (ROS) through Superoxide Dismutase 1 (SOD1). Dysfunctional mitochondria, manifesting decreased activity of electron transport chain (ETC) complexes and high ROS levels, are involved in Alzheimer’s disease (AD) pathogenesis. We hypothesized that neuronal mitochondrial dysfunction in AD is reflected in ETC and SOD1 levels and activity in plasma neuron-derived extracellular vesicles (NDEVs). We immunoprecipitated NDEVs targeting neuronal marker L1CAM from two cohorts: one including 22 individuals with early AD and 29 control subjects; and another including 14 individuals with early AD and 14 control subjects. In the first cohort, we measured levels of complexes I, III, IV, ATP synthase, and SOD1; in the second cohort, we measured levels and catalytic activity of complexes IV and ATP synthase. AD individuals had lower levels of complexes I (*p* < 0.0001), III (*p* < 0.0001), IV (*p* = 0.0061), and V (*p* < 0.0001), and SOD1 (*p* < 0.0001) compared to controls. AD individuals also had lower levels of catalytic activity of complex IV (*p* = 0.0214) and ATP synthase (*p* < 0.0001). NDEVs confirm quantitative and functional abnormalities in ECT complexes and SOD1 previously observed in AD models and during autopsy, opening the way for using them as biomarkers for mitochondrial dysfunction in AD.

## 1. Introduction

A wide range of structural and functional mitochondrial abnormalities are found in Alzheimer’s disease (AD) (for a comprehensive review, see [1]). The main function of mitochondria is to generate energy, mainly through oxidative phosphorylation coupled to electron transfer across the respiratory or electron transfer chain (ETC), which consists of complexes I through IV, and ATP synthase. In addition to production of water by complete reduction of oxygen—the final recipient of electrons in the ETC—partial reduction of oxygen may occur, generating potentially cytotoxic reactive oxygen species (ROS). The brain is especially susceptible to oxidative injury because of its high consumption of oxygen and low levels of endogenous antioxidants. Oxidative injury of brain cells by lipid peroxidation and nucleic acid oxidation is a prominent neurodegenerative mechanism [2]. Studies investigating the mechanisms of oxidative injury in the AD brain have largely focused on abnormalities of superoxide dismutase (SOD), a mitochondrial enzyme that eliminates ROS [3]. The number of SOD-positive neurons is decreased in the hippocampus and frontotemporal cortex of AD patients [4]. Interestingly, brain areas with lower density of neurofibrillary tangles (the hallmark abnormal tau deposits in AD) and milder neuronal loss compared to more severely affected areas, also show normal total SOD activity, suggesting that SOD activity may be counteracting tau aggregation and neurodegenerative processes [5].

AD, like other neurodegenerative diseases, is the product of a series of inter-related pathogenic processes that develop over decades. To study these processes in living individuals, we and others have been developing biomarkers utilizing blood extracellular vesicles (EVs), especially a selectively immunocaptured sub-population of neuronal origin (NDEVs) [6,7], as a form of “liquid biopsy”. In addition to biomarkers reflecting amyloidosis, tauopathy, neurodegeneration, and insulin resistance [6,8,9,10,11], EVs and NDEVs contain mitochondrial cargo, including mitochondrial RNAs [12], that may be used to investigate mitochondrial abnormalities in AD. The study of abnormalities of mitochondrial oxidative phosphorylation and ROS elimination in living humans has recently become possible through assessing plasma NDEV levels of ETC proteins and SOD1, a pursuit initially undertaken in relation to psychosis [13,14].

In this study, we investigated SOD1, mitochondrial ETC complex proteins, and ATP synthase in individuals with AD as well as control subjects by leveraging NDEVs. The dysfunctional pattern of neuron mitochondrial electron transfer proteins in AD may be expected to result in increased production of ROS and decreased production of ATP [15]. Therefore, we hypothesized that NDEVs of individuals with AD compared to controls have decreased levels and activity of ETC complexes, ATP synthase and SOD1, a pattern that was demonstrated by this study.

## 2. Materials and Methods

### 2.1. Participants

We carried out our study in two cohorts. The first cohort consisted of 22 individuals with high-probability early AD according to the NIA-AA and IWG-2 criteria [16] who had been evaluated extensively in the Clinical Research Unit of the U.S. National Institute on Aging (NIA; Baltimore, MD, USA); and 29 healthy, cognitively normal controls who had donated blood at the University of California, San Francisco (UCSF)-affiliated Jewish Home of San Francisco (JHSF) in the same time period as the patients. Demographics and clinical data for the two cohorts are summarized in Table 1. Individuals with AD had abnormal CSF levels of amyloid β-peptide (Aβ) 1–42 (Aβ42) < 192 pg/mL and of P-T181-tau > 23 pg/mL that supported their diagnosis [17]. Due to limited plasma from controls in the first cohort, we analyzed a second cohort including a subset of 14 individuals with high-probability early AD from the previous cohort of patients and a new set of 14 age- and sex-matched healthy, cognitively normal controls from NIA clinical studies (also Table 1). Performance and procedures of all studies were approved by the NIH (Protocols 10AG0423, approved through 27 October 2022; and 03-AG-0325, approved through 5 October 2022) or for the JHSF by the UCSF Institutional Review Boards (Protocol 11-05562, approved through 25 July 2022) and all participants signed approved Consent Forms.

### 2.2. Isolation of Neuron-Derived EVs (NDEVs)

Aliquots of 0.25 mL plasma were incubated with 0.1 mL of thromboplastin D (ThermoFisher Scientific, Waltham, MA, USA) for 30 min at room temperature, followed by addition of 0.15 mL of calcium- and magnesium-free Dulbecco’s balanced salt solution (DBS) with protease inhibitor cocktail (Roche, Indianapolis, IN, USA) and phosphatase inhibitor cocktail (Thermo Fisher Scientific; DBS++) as described in [7,18]. After centrifugation at 3000× *g* for 30 min at 4 °C, total particles were precipitated with 126 μL per tube of ExoQuick (System Biosciences, Mountain View, CA, USA) and centrifugation at 1500× *g* for 30 min at 4 °C. To isolate the enriched subpopulation of NDEVs, total EVs were resuspended in 0.35 mL of DBS and incubated for 60 min at room temperature with 2.0 μg of mouse anti-human CD171 (L1CAM neural adhesion protein) biotinylated antibody (clone 5G3; eBiosciences, San Diego, CA, USA) in 50 μL of 3% bovine serum albumin (BSA; 1:3.33 dilution of Blocker BSA 10% solution in DBS; ThermoFisher Scientific) per tube with mixing for 60 min, followed by addition of 10 μL of streptavidin agarose Ultralink resin (ThermoFisher Scientific, Waltham, MA, USA) in 40 μL of 3% BSA and incubation for 30 min at room temperature with mixing. After centrifugation at 800× *g* for 10 min at 4 °C and removal of the supernatant, each pellet was suspended in 100 μL of cold 0.05 M glycine-HCl (pH 3.0) by gentle mixing for 10 s and centrifuged at 4000× *g* for 10 min, all at 4 °C. Supernatants then were transferred to clean tubes containing 25 μL of 10% BSA and 10 μL of 1 M Tris-HCl (pH 8.0) and mixed gently. An aliquot of 5 μL was removed from each tube for EV counts before addition of 370 μL of mammalian protein extraction reagent (M-PER; ThermoFisher Scientific, Waltham, MA, USA). Resultant 0.5 mL lysates of NDEVs were stored at −80 °C.

### 2.3. NDEV Characterization

To determine size and number of NDEVs, each 5 µL suspension was diluted 1:50 in PBS and suspensions were determined by nanoparticle tracking analysis (NTA) using the Nanosight NS500 system with a G532 nm laser module and NTA 3.1 nanoparticle tracking software (Malvern Instruments, Malvern, UK). Camera settings were as follows: gain 366; shutter 31.48; and frame rate 24.9825 frames/s. Brownian motion was captured by performing 5 repeated 60 s video recordings.

The protein concentrations of lysed NDEVs were quantified with the Pierce Coomassie reagent (ThermoFisher Scientific, Waltham, MA, USA) according to the manufacturer’s manual. For Western Blotting, samples were resolved on NuPAGE 4–12% Bis-Tris gels (ThermoFisher Scientific, Waltham, MA, USA). Proteins were transferred using iBlot 2 on PVDF membranes (ThermoFisher Scientific, Waltham, MA, USA). Membranes were blocked with 5% non-fat milk in TBS-T for 1 hr at RT. Membranes were incubated with Alix (1:1000; NBP1-90201; Novus Biologicals, Centennial, CO, USA), CD9 (1:500; #312102; Biolegend, San Diego, CA, USA), GM130 (Abcam, Inc., Cambridge, MA, USA; ab52649: 1:1000), and ApoA1 (R&D Systems, Minneapolis, MN, USA; AF3664; 1:500) overnight at 4 °C. Anti-mouse, anti-Rabbit, and anti-Goat HRP conjugated secondary antibodies (Cell Signaling, Danver, MA, USA; 1:3000) were used. Images were captured with Sapphire Biomolecular Imager (Azure Biosystems, Dublin, CA USA) using Amersham ECL Prime Western Blotting Detection Reagent (GE Healthcare, Silver Spring, MD, USA).

### 2.4. Quantification of NDEV Proteins

NDEV proteins were quantified by enzyme-linked immunosorbent assay (ELISA) kits for human tetraspanin exosome marker CD81, SOD1 (superoxide dismutase 1)(Ray Biotech, Norcross, GA, USA), subunit 1 of complex I (NADH-ubiquinone oxidoreductase)(DL-Develop Corp. by American Research Products; Waltham, MA, USA), subunit 6 of complex I (NADH-ubiquinone oxidoreductase)(Cusabio Technology by American Research Products, Waltham, MA, USA), subunit 10 of complex III (cytochrome b-c1 oxidase) (Abbkine, Inc. by American Research Products; Waltham, MA, USA), subunit 1 of complex IV (cytochrome C oxidase)(Cloud-Clone Corp by American Research Products Waltham, MA, USA), and ATP synthase (Abcam, Inc., Cambridge, MA, USA). The mean value for CD81 in each group was set at 1.00, and relative values of CD81 for each sample were used to normalize their recovery.

### 2.5. Measurement of ATP Synthase and Complex IV Activity

To explore functional correlates of differences in concentration, we sought to investigate the activity of complex IV and ATP synthase in NDEVs (prioritizing ATP synthase activity given limited sample availability for the first cohort). For the first cohort, ATP synthase activity in NDEVs was measured using a commercial assay kit (Abcam, Inc., Cambridge, MA, USA; ab109714), in which ATP synthase was immunocaptured and its catalytic activity based on conversion of NADH to NAD+ was quantified. The mean values for ATP synthase activity in the first cohort were normalized to values of CD81.

To ensure that results were robust to the method of normalization and determine specific activity per amount of protein, in the second cohort, ATP synthase activity in NDEVs was measured using a slightly different assay (Abcam, Inc., Cambridge, MA, USA; ab109716). In this assay, immunocaptured ATP synthase was first measured for its NADH to NAD+ conversion activity, then followed by ELISA to measure its quantity in the same assay kit wells. The mean values for all determinations of ATP synthase activity were then normalized to values of ATP synthase quantity.

Due to limited sample availability, complex IV activity was measured only in the second cohort using a commercial assay (Abcam, Inc., Cambridge, MA, USA; ab109910). In this assay, the activity of immunocaptured complex IV was first measured for oxidation of reduced cytochrome c, then followed by ELISA for its quantity in the same samples. The mean values for all determinations of complex IV activity were then normalized to values of complex IV quantity.

## 3. Results

### 3.1. NDEV Characterization

**Figure 1:**
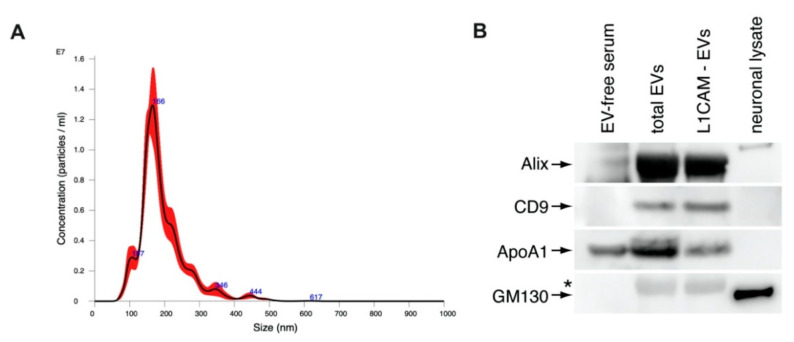
(**A**) NTA analysis of the size distribution of L1CAM-immunoprecipitated NDEVs. (**B**) Immunoblots of Alix, CD9, ApoA1, and GM130 in total EVs and NDEV samples. * Nonspecific band.

Mean ± S.E.M. concentration of NDEVs in immuno-selected suspensions were 137 ± 5.16 × 10^9^/mL for controls and 132 ± 4.78 × 10^9^/mL for AD individuals. NDEVs of controls and AD individuals were of similar diameters ranging from 70 to 118 nm. CD81 levels showed no differences between controls and AD individuals at 1250 ± 126 pg/mL and 1532 ± 78.9 pg/mL, respectively. The neuronal marker neuron-specific enolase measured in NDEVs extracts confirmed a high degree of neuronal cargo enrichment, with a mean ± S.E.M. of 5816 ± 142 pg/mL in controls and 6049 ± 153 pg/mL in AD individuals, more than ten-fold higher than levels previously observed in astrocyte-derived exosome extracts [19]. Representative NTA analysis profile of NDEVs shows a size distribution typical of exosomes and microvesicles (Figure 1A). Immunoblotting analysis of NDEVs shows the enrichment of EV markers Alix and CD9, confirming that isolated particles are EVs (Figure 1B). Furthermore, the reduction in Apolipoprotein A1 shows that NDEV isolation by immunoprecipitation against L1CAM reduced lipoprotein particles in EV samples and Golgi marker GM130 (Figure 1B).

### 3.2. NDEV Mitochondrial Proteins

**Figure 2:**
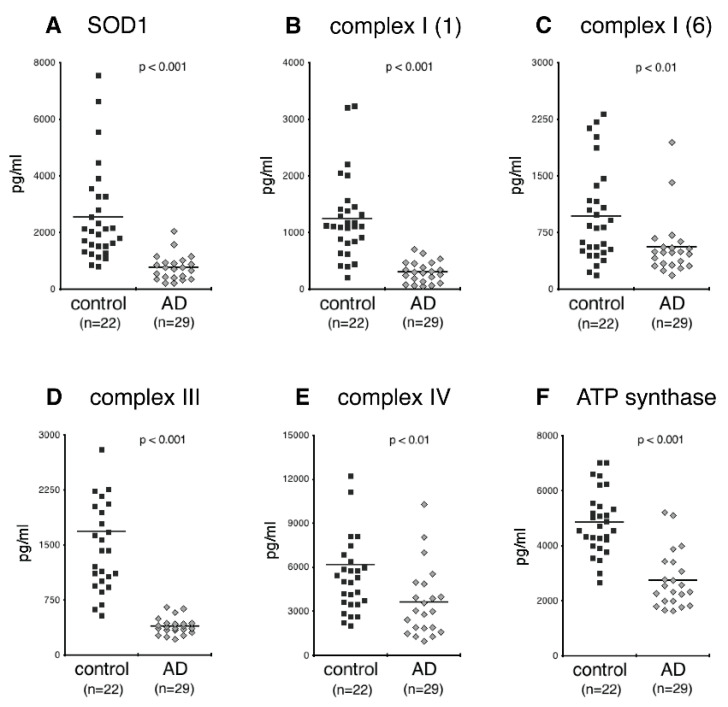
Levels of SOD1 and constituents of the mitochondrial oxidative phosphorylation system in plasma NDEVs of control and AD. Each data point represents the value for one study participant. The mean ± S.E.M. of control and AD groups, respectively, were 2252 ± 313 pg/mL and 769 ± 96 pg/mL, *p* < 0.001, for SOD1 (**A**); 1254 ± 135 pg/mL and 306 ± 40 pg/mL, *p* < 0.001, for subunit 1 of complex I (**B**); 972 ± 116 pg/mL and 558 ± 85 pg/mL, *p* < 0.01, for subunit 6 of complex I (**C**); 1692 ± 153 pg/mL and 399 ± 24 pg/mL, *p* < 0.001, for subunit 10 of complex III (**D**); 6209 ± 752 pg/mL and 3647 ± 513 pg/mL, *p* < 0.01, for subunit 1 of complex IV (**E**); 4863 ± 213 pg/mL and 2762 ± 221 pg/mL, *p* < 0.001, for ATP synthase (**F**). All values were normalized for content of the exosome marker CD81. Statistical significance of differences in values between control and AD groups were calculated by two sample t tests.

CD81-normalized NDEV levels of SOD1 levels were lower in AD individuals than controls (Figure 2A; *p* < 0.001). CD81-normalized NDEV levels of complexes I (subunits 1 and 6), III and IV, as well as ATP synthase (complex V) all were significantly lower in AD than controls (Figure 2B–F; for complexes I, III, and V, *p* < 0.0001; for complex IV, *p* = 0.0061).

**Figure 3:**
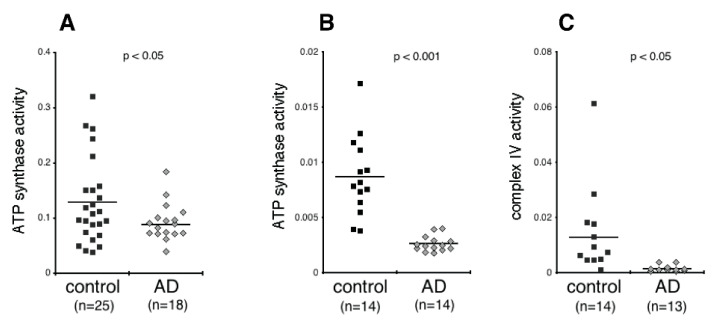
Activity of the mitochondrial oxidative phosphorylation proteins in plasma NDEVs of control and AD. Each data point represents the value for one study participant. (**A**), cohort 1; (**B**,**C**), cohort 2. The numbers of study participants are indicted on the graphs. The mean ± S.E.M. of control and AD groups, respectively, was 0.129 ± 0.015 and 0.088 ± 0.009, *p* < 0.05, for ATP synthase, cohort 1 (**A**); 0.009 ± 0.001 and 0.003 ± 0.0002, *p* < 0.001, for ATP synthase, cohort 2 (**B**); 0.013 ± 0.004 and 0.001 ± 0.0003, *p* < 0.05, for complex IV (**C**). Values in (**A**) were normalized for content of the EV marker CD81. Values in (**B**,**C**) were normalized for quantity of the respective protein that was determined within the same assay (see Methods). Statistical significance of differences in values between control and AD groups were calculated by two sample *t* tests.

We next measured the activity of ATP synthase and complex IV. As shown in Figure 3A,B, the activity of ATP synthase was consistently lower in AD than controls in both cohorts (*p* < 0.05, *p* < 0.001, respectively). Moreover, this result held whether activity was normalized by CD81 (i.e., activity per EV cargo) in the first cohort, or by quantity in the same assay kit wells (i.e., activity per amount of ATP synthase). The activity of complex IV in the NDEVs (which was evaluated in the second cohort and was normalized by quantity in the same assay kit wells) was also significantly lower in AD than controls (Figure 3C; *p* < 0.05).

## 4. Discussion

Over the past year it has become clear that EVs contain mitochondrial components ranging from DNA and RNA to proteins [12,19,20,21]. We now recapitulate this conclusion in the context of NDEVs from the plasma of AD patients. Specifically, we demonstrate the presence of mitochondrial ETC proteins, ATP synthase, and SOD1 in plasma NDEVs and show that levels of these mitochondrial proteins in NDEVs are distinctly different between AD and control subjects. Furthermore, we assessed the activities of some of the electron transport chain proteins, complexes IV and ATP synthase, and demonstrate decreased catalytic activity of ATP synthase and complex IV in NDEVs of AD patients. We hope that our study opens a way for bridging the translational gap between mitochondrial biology, typically studied in vitro, ex vivo, and in animal models, and clinical studies in AD.

Our findings are largely consistent with several recent studies showing mitochondrial proteins in EVs. In EVs isolated from neuronal cell line SH-SY5Y and human plasma, mitochondrial matrix protein citrate synthase and mitochondrial outer membrane protein VDAC are detectable [21]. Brain tissue derived EVs from Down syndrome human and mouse models are enriched for all ETC complexes [19]. Moreover, proteomic analysis of EVs isolated from the media of cultured rodent hippocampal neurons show the presence of multiple subunits of several ETC complexes [20]. The findings of this study agree with a growing body of literature that implicates structural and functional mitochondrial abnormalities in AD [1]. The pattern of low levels of complexes I, III, IV, and ATP synthase observed here has already been documented in a proteomic study of frozen brain tissues of AD patients and controls, which revealed decreased levels of complexes I, III and ATP synthase early in the course of AD, although decreased levels of IV were only seen at later stages of AD [15]. Interestingly, we found complex IV level to be depressed, but not as depressed as those of complex I and III, or ATP synthase; given that these findings were derived from comparing control subjects with individuals with early AD, the relative preservation of complex IV is consistent with these earlier findings [15]. A relative imbalance between higher complex IV levels and more diminished complex V levels putatively favors partial rather than complete O_2_ reduction and ROS generation. Intriguingly, an early study showed that neurons demonstrating increased oxidative damage in AD have a striking and significant increase in cytochrome oxidase [22]. Moreover, the mitochondrial level of complex IV has been linked functionally to low synaptic connectivity [23], which is increasingly being identified as a core feature of AD pathogenesis [24]. miRNA-mediated knockdown of complex IV in cultured rat primary neurons and in vivo in rats results in lower levels of inhibitory and excitatory synaptic markers with concomitant decreases in synaptic connectivity [23]. Moreover, the present study readily quantified catalytic activity for various ETC complexes in NDEVs, whereas others have been unable to do so in total plasma EVs [21]. Whether the NDEV immunoselection protocol followed here provided us with higher levels of intact proteins compared to other methodologies should be clarified by future research. Finally, the decrease in SOD1 levels seen in NDEVs is consistent with results of analyses of SOD1 levels in brain tissues of animal models of AD [25,26].

Aggregating proteins involved in AD, Parkinson’s disease, and other neurodegenerative diseases are induced by and interact with ROS [27,28]. Specifically, neurotoxic Aβ peptides decrease levels and activities of complexes I, II, and IV and enhance generation of ROS, whereas, reciprocally, ROS stimulates production of Aβ [1,29,30]. Additionally, Aβ colocalizes with the α-subunit of ATP synthase in the hippocampus and cortex of AD mice models and this interaction has been also shown in the plasma membrane, which results in reduced extracellular ATP levels leading to reduced long-term potentiation (LTP) and increased long-term depression (LTD) at synapses [31]. Moreover, inhibiting oxidative metabolism promotes amyloidogenic cleavage of amyloid precursor protein (APP) [1,32]. Aβ and ROS synergistically damage synapses, activate microglia and neuroinflammation, and impair functions of the cerebral microvasculature and the blood brain barrier [1,30,33]. In human cultured neural cells, cytoplasmic SOD1 binds to intracellular Aβ forming perinuclear complexes manifesting reduced SOD1 activity [34]. In AD mouse models, SOD1 deficiency promotes Aβ oligomerization and cognitive/behavioral impairment, whereas SOD1 supplementation decreases progression of AD pathology and improves cognition [35,36].

Previous research has shown that mitochondria may act as sinks and sites of degradation for aggregation-prone proteins [1]. Diminished levels and activities of mitochondrial proteins in NDEVs may correspond to decreases in functional mitochondria in their originating neurons favoring pathogenic protein aggregation. On the other hand, Aβ and tau pathologies affect mitochondrial dynamics. In a mouse model of AD, tau aggregation disrupts the distribution of mitochondria to neurites leading to axonal and synaptic degeneration; this pattern is also observed in post-mortem brain samples from AD individuals [37]. Furthermore, hyperphosphorylated tau can disrupt the balance between mitochondrial fission and fusion leading to synaptic degeneration [38].

In a previous study, we demonstrated the presence of all mitochondrial RNAs (tRNAs, mRNAs, and rRNAs) in circulating total EVs and showed (by RNA sequencing confirmed by RT-qPCR) that individuals with AD have higher levels of all mitochondrial RNAs compared to controls [12]. Findings were further confirmed for NDEVs from cultured cells challenged by Aβ [12], although the need for high RNA levels for reliable RNA sequencing prevented us from examining mitochondrial RNAs in circulating NDEVs. Since then, the presence of multiple mitochondrial RNAs in EVs has been confirmed by others [21]. Intriguingly, the direction of difference observed for RNAs is the opposite from the one observed for mitochondrial proteins in the present study. This raises the possibility that different mitochondrial molecules may be sorted differently into EVs (or even into different EV subclasses), representing true cargo and not contaminant [21]. It is tempting to speculate that RNAs located in the mitochondrial matrix may be sorted out differently into endosomes than ETC and SOD1 proteins and that these mechanisms may be differentially affected by AD. In that regard, we recently demonstrated that neuronal mitochondria produce single and double membrane-containing protrusions of various morphologies, likely to result in the generation of vesicles [39]. These vesicles reach other vesicular intracellular compartments likely including the lysosome and recycling and late endosome [39], which is the site of biogenesis of exosomes. Although we do not readily ascribe to the use of the term “mitovesicles” to refer to a putative distinct sub-population of double-membranous EVs [19], since the term may create confusion with vesicles generated by mitochondria and that remaining intracellular [39], the presence of mitochondrial cargos in EVs is beyond doubt [21].

The main limitations of this study are the facts that it involved two rather small cohorts and only included patients with early AD (phenotypically, at the MCI and mild dementia stages). Therefore, conclusions may not generalize to other stages of the disease and findings should be replicated in larger cohorts spanning the entire AD pathogenic spectrum. However, this study was based on high-quality cohorts, since it included patients meeting criteria for high-probability AD based on CSF biomarkers, whereas controls (at least in the second cohort) have been followed longitudinally and have maintained normal cognitive status over time. Moreover, we view the fact that findings were consistent across two cohorts with different ranges of MMSE and ADAS-cog as a strength, since it suggests that abnormalities may occur across a wide range of clinical AD. Furthermore, the current study was limited in the scope of mitochondrial abnormalities with relevance to AD pathogenesis it examined. However, we hope that our work will serve as a primer for many broad-scoped and in-depth studies in understanding mitochondrial abnormalities in AD and Parkinson’s disease. For instance, future studies may wish to investigate the presence of the nuclear factor erythroid 2-related factor (NRF2/Nrf2) in NDEVs, since this ‘master regulator of cellular anti-oxidant response’ may be critical in mitigating AD pathogenic cascades [40], whereas it shows nuclear localization associated with the development of a-synuclein pathology in Parkinson’s disease [41].

It is already widely accepted that a conceptualization of AD as amyloidosis, tauopathy, and neurodegeneration (ATN) is overly restrictive; the incorporation of mitochondrial biomarkers into the canon of AD biomarkers may further improve its overall reliability for diagnosis and monitoring of disease activity across different stages of AD. Ultimately, in vivo detection and tracking of mitochondrial dysfunction with NDEVs may improve our understanding of the role metabolic abnormalities play in the evolution of AD and may help identify subgroups of AD patients for whom mitochondrial abnormalities are particularly important. Such individuals may be selected for clinical trials of the evolving therapeutic approaches that intend to rectify mitochondrial and other metabolic abnormalities in AD [42], bringing us even closer to the development of precision medicine for the disease.

## Figures and Tables

**Table 1:** Demographics and clinical data.

	First Cohort		Second Cohort	
	Control	AD	Control	AD
Age	73.4 ± 2.11	73.2 ± 1.65	73.1 ± 2.11	73.1 ± 2.36
Sex (F/M)	13/9	17/12	7/7	7/7
MMSE	28–30	23–26	-	18–30
ADAS-Cog (21−23)	0–5	10–16	-	5–25
CDR	0.0	0.5–1.0	-	0.5–1.0

## Data Availability

The data presented in this study are available on request from the corresponding author.
